# Supplementary material for: From sequence to enzyme mechanism using multi-label machine learning
Source: BMC Bioinformatics. 2014 May 19;15:150. doi: 10.1186/1471-2105-15-150 (PMC4229970; doi:10.1186/1471-2105-15-150)
Supplement: Additional file 2 — Java code of ml2db. Additional file ml2db_code.tar.gz contains the Java source code to run the multi-label machine learning experiments and save the results to database. The code’s Javadoc is included. [file 1471-2105-15-150-S2.zip › additional file 2/ml2db/ml2db/doc/mulan/evaluation/measure/package-summary.html]

mulan.evaluation.measure


JavaScript is disabled on your browser.


- Overview
- Package
- Class
- Use
- Tree
- Deprecated
- Index
- Help

- Prev Package
- Next Package

- Frames
- No Frames

- All Classes

# Package mulan.evaluation.measure

- Interface Summary

  | Interface | Description |
  | --- | --- |
  |  |  |
  | --- | --- |
  | MacroAverageMeasure | Interface for macro average measures |
  | Measure | Interface for a measure, used to evaluate the performance of a multi-label learner on when performing multi-label learning task. |
- Class Summary

  | Class | Description |
  | --- | --- |
  |  |  |
  | --- | --- |
  | AveragePrecision | Implementation of the average precision measure. |
  | BipartitionMeasureBase |  |
  | ConfidenceMeasureBase |  |
  | Coverage | Implementation of the coverage measure. |
  | ErrorSetSize | Measure based on the ErrorSetSize ranking loss function |
  | ExampleBasedAccuracy | Implementation of the example-based accuracy measure. |
  | ExampleBasedBipartitionMeasureBase | Base class for example-based bipartition measures |
  | ExampleBasedFMeasure | Implementation of the example-based F measure. |
  | ExampleBasedPrecision | Implementation of the example-based precision measure. |
  | ExampleBasedRecall | Implementation of the example-based recall measure. |
  | ExampleBasedSpecificity | Implementation of the example-based recall measure. |
  | GeometricMeanAverageInterpolatedPrecision | Implementation of GMAiP (Geometric Mean Average Interpolated Precision) |
  | GeometricMeanAveragePrecision | Implementation of GMAP (Geometric Mean Average Precision) |
  | HammingLoss | Implementation of the Hamming loss function. |
  | HierarchicalLoss | Implementation of the Hierarchical loss measure. |
  | InformationRetrievalMeasures | Class for computing various information retrieval measures. |
  | IsError | Measure based on the IsError ranking loss function |
  | LabelBasedAUC | Implementation of the label-based macro precision measure. |
  | LabelBasedAveragePrecision |  |
  | LabelBasedBipartitionMeasureBase | Base class for label-based bipartition measures |
  | LabelBasedFMeasure | Base implementation of the label-based macro/micro f-measures. |
  | LabelBasedPrecision | Base implementation of the label-based macro/micro precision measures. |
  | LabelBasedRecall | Common class for the micro/macro label-based recall measures. |
  | LabelBasedSpecificity | Common class for the micro/macro label-based recall measures. |
  | LossBasedBipartitionMeasureBase |  |
  | LossBasedRankingMeasureBase |  |
  | MacroAUC | Implementation of the macro-averaged AUC measure. |
  | MacroFMeasure | Implementation of the macro-averaged f measure. |
  | MacroPrecision | Implementation of the macro-averaged precision measure. |
  | MacroRecall | Implementation of the macro-averaged recall measure. |
  | MacroSpecificity | Implementation of the macro-averaged recall measure. |
  | MeanAverageInterpolatedPrecision | Implementation of MAiP (Mean Average Interpolated Precision) |
  | MeanAveragePrecision | Implementation of MAP (Mean Average Precision) |
  | MeasureBase |  |
  | MicroAUC | Implementation of the micro-averaged AUC measure. |
  | MicroFMeasure | Implementation of the micro-averaged precision measure. |
  | MicroPrecision | Implementation of the micro-averaged precision measure. |
  | MicroRecall | Implementation of the micro-averaged recall measure. |
  | MicroSpecificity | Implementation of the micro-averaged recall measure. |
  | OneError | Measure based on the one error loss function |
  | RankingLoss | Implementation of the ranking loss measure. |
  | RankingMeasureBase |  |
  | SubsetAccuracy | Implementation of the subset accuracy measure. |

- Overview
- Package
- Class
- Use
- Tree
- Deprecated
- Index
- Help

- Prev Package
- Next Package

- Frames
- No Frames

- All Classes
